# Defining the Indications and Levels of Erector Spinae Plane Block in Pediatric Patients: A Retrospective Study of Our Current Experience

**DOI:** 10.7759/cureus.5348

**Published:** 2019-08-08

**Authors:** Can Aksu, Yavuz Gurkan

**Affiliations:** 1 Anesthesiology, Kocaeli University, Kocaeli, TUR; 2 Anesthesiology, Koç University, İstanbul, TUR

**Keywords:** erector spinae plane block, postoperative pain, pediatric anesthesia, postoperative analgesia, ultrasound

## Abstract

Aim

The primary aim of this study was to evaluate the indications, effectiveness, application levels, and local anesthetic (LA) dosages used in erector spinae plane block (ESPB) in pediatric patients  based on our clinical data. The secondary aim was to compare previously reported pediatric ESPBs with our data and to prepare a mini-guide for future clinical applications.

Materials and methods

One hundred and forty-one pediatric patients who received ESPB and were operated by the Department of Pediatric Surgery were included in this retrospective observational study. ESPB is routinely performed with 0.5 ml/kg 0.25% bupivacaine (max 20 ml). Demographic data and the type of surgery were recorded. Face, Legs, Activity, Cry, and Consolability (FLACC) or Numeric Rating Scale (NRS) scores, analgesic requirements, and the type of analgesic administered at postoperative period were recorded.

Results

ESPB was applied using three different techniques, the classic approach, the transverse approach, and the Aksu approach. Unilateral ESPB was performed on 112 patients, while 29 received a bilateral block. ESPB used for 13 different indications.

Conclusion

ESPB is a relatively safe and effective procedure for achieving opioid-free postoperative analgesia in many different surgical procedures in pediatric patients.

## Introduction

The erector spinae plane block (ESPB) was first described by Forero et al. [1] in 2016 and has since become a trending topic among clinicians. However, the problem with ESPB is that there still is a lack of knowledge concerning its mechanism of action, and clinical reports/studies have produced inconsistent results regarding local anesthetic (LA) distribution [2-4]. However, although the mechanism of action and drug distribution have not been clearly elucidated, the clinical significance of this block is undeniable.

There have been a few randomized prospective studies of ESPB in terms of its clinical effectiveness, but all, with one exception, have involved adults [5-7]. Pediatric case reports are also limited compared to adults [8-24]. We have been applying ESPB almost since it was first described. However, ESPB applications for pediatric patients in our clinic commenced following a learning curve. Nonetheless, we have to date performed numerous ESPB applications in pediatric patients with different indications.

Various reviews and reports have investigated the indications for ESPB in adults, but to the best of our knowledge, there is no published definitive study for pediatric patients. We have been using standardized methods for perioperative analgesia management of pediatric patients in our clinic, and the data are routinely preserved. The primary aim of this study was to evaluate the indications, effectiveness, application levels, and LA dosages used in ESPB in pediatric patients based on our clinical data. The secondary aim was to compare previously reported pediatric ESPBs with our data and to prepare a mini-guide for future clinical applications.

## Materials and methods

This retrospective cohort study was conducted after receipt of approval from the Local Ethics Committee (GOKAEK 2019/85) and registration with clinicaltrials.org (NCT03906019). The study was conducted in accordance with the Declaration of Helsinki. We reviewed all medical records of patients operated by the Department of Pediatric Surgery between September 1, 2017 and March 1, 2019, and undergoing ESPB as a part of postoperative analgesia management. Following the establishment of the patient population, other requested data elements were retrieved in a series of further queries and recorded on IBM SPSS for Windows® version 20.0 (SPSS, Chicago, IL, USA) software for subsequent analysis. Collected data included demographic information, patient position for the block, indication/surgery type, level of ESPB, unilateral or bilateral application, the total volume applied, additional analgesic use, kind of analgesic used, if applicable, and any additional descriptive data the patient provided during follow-up.

Our clinic has a standard protocol for pediatric surgeries for both anesthesia and pain management. Every patient under the age of 15 years receives ESPB under general anesthesia before the start of surgery, and patients older than 15 years were asked whether or not they are willing to undergo awake block application. We use 0.25% bupivacaine as the LA for all block applications. The volume of LA administered for a unilateral block is 0.5 ml/kg (max 20 ml). Every patient receives paracetamol 15 mg/kg iv routinely at the end of surgery. For the postoperative follow-up, except in day-case surgery, paracetamol 15 mg/kg is routinely administered to every patient under the age of one year every six hours for the first postoperative day. In subsequent days, Face, Legs, Activity, Cry, and Consolability (FLACC) or Numeric Rating Scale (NRS) scores are used for administering any analgesic drugs. There is no routine analgesic regimen for children over one year, although additional analgesic drug requirements are decided based on FLACC or NRS scores for the entire postoperative period. FLACC is the preferred scoring system for patients up to seven years of age, while the NRS score is the accepted pain evaluation method for patients older than seven. Paracetamol, non-steroidal anti-inflammatory drugs or tramadol are used depending on pain severity. A pediatric pain nurse is responsible for all pain evaluations, recording pain score data, and administering analgesic drugs.

For day case surgeries, patients are discharged home after a six-hour follow-up period. In order to assess pain and administer analgesic drugs at home, pain-scoring systems are taught to parents, who are instructed to administer 15 mg/kg oral acetaminophen as the first choice rescue analgesia and 7 mg/kg oral ibuprofen if the pain is severe. Analgesic requirements and the type of analgesic administered were questioned and recorded at postoperative follow-up visits for all patients. The acute pain team routinely maintains all data. Due to this standardization, the results of this study represent retrospective analyses of routinely collected data.

## Results

Data for 141 pediatric patients receiving ESPB, 44 girls and 97 boys, were included in the study. The mean age of the patients was 4.95 ± 4.09 years (min 0.25 - max 17), and their mean weight was 21.58 ± 14.86 kg (min 4 - max 68). The American Society of Anesthesiology (ASA) physical status scores were '1' for 127 patients and '2' for 14. Mean operative time was 65.32 ± 35.91 min (min 30 - max 300).

**Figure 1:**
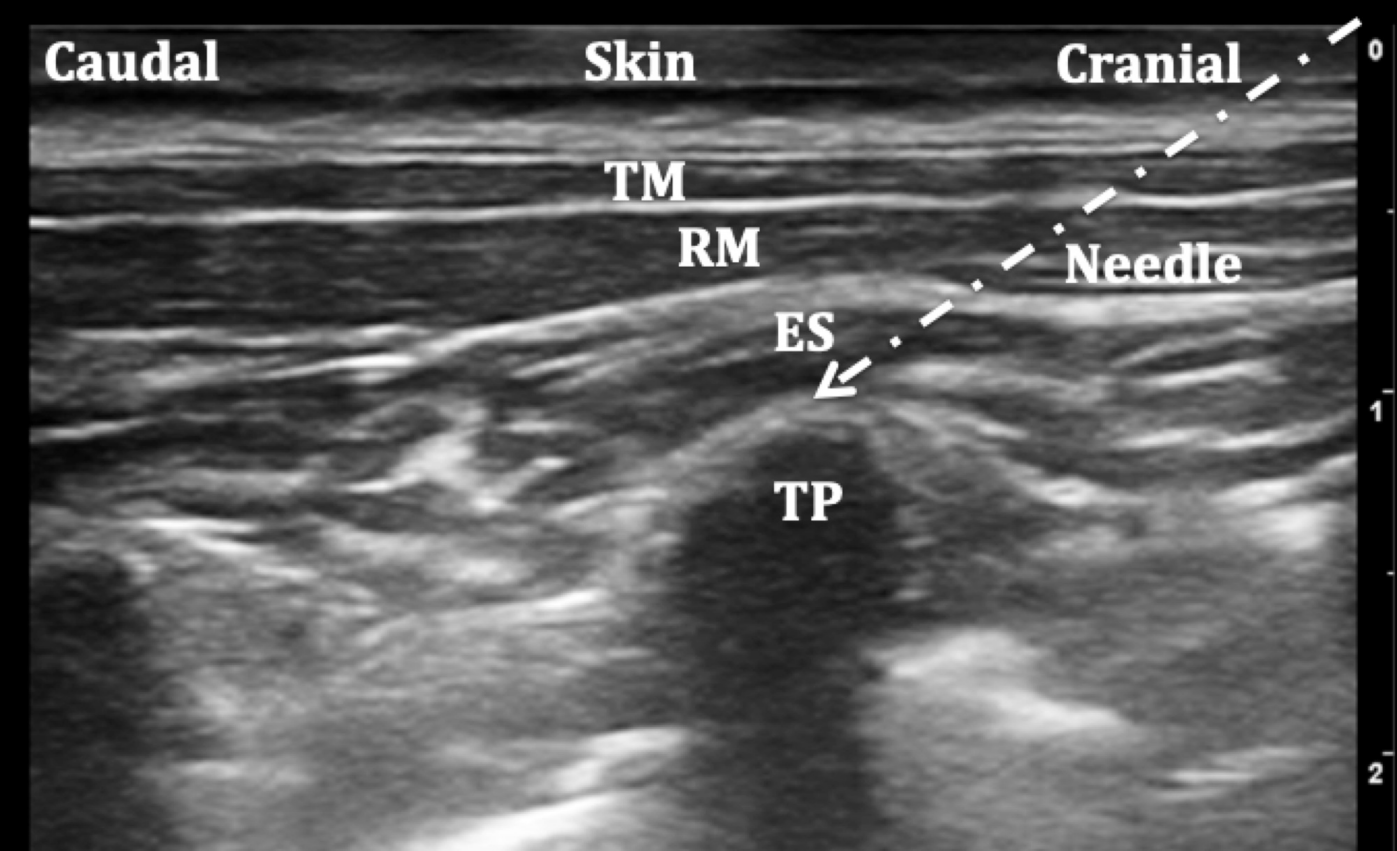
Ultrasound image of the classic approach to erector spinae plane block (ESPB) TM: Trapezius muscle, RM: Rhomboid muscle, ES: Erector spinae, TP: Transverse process.

**Figure 2:**
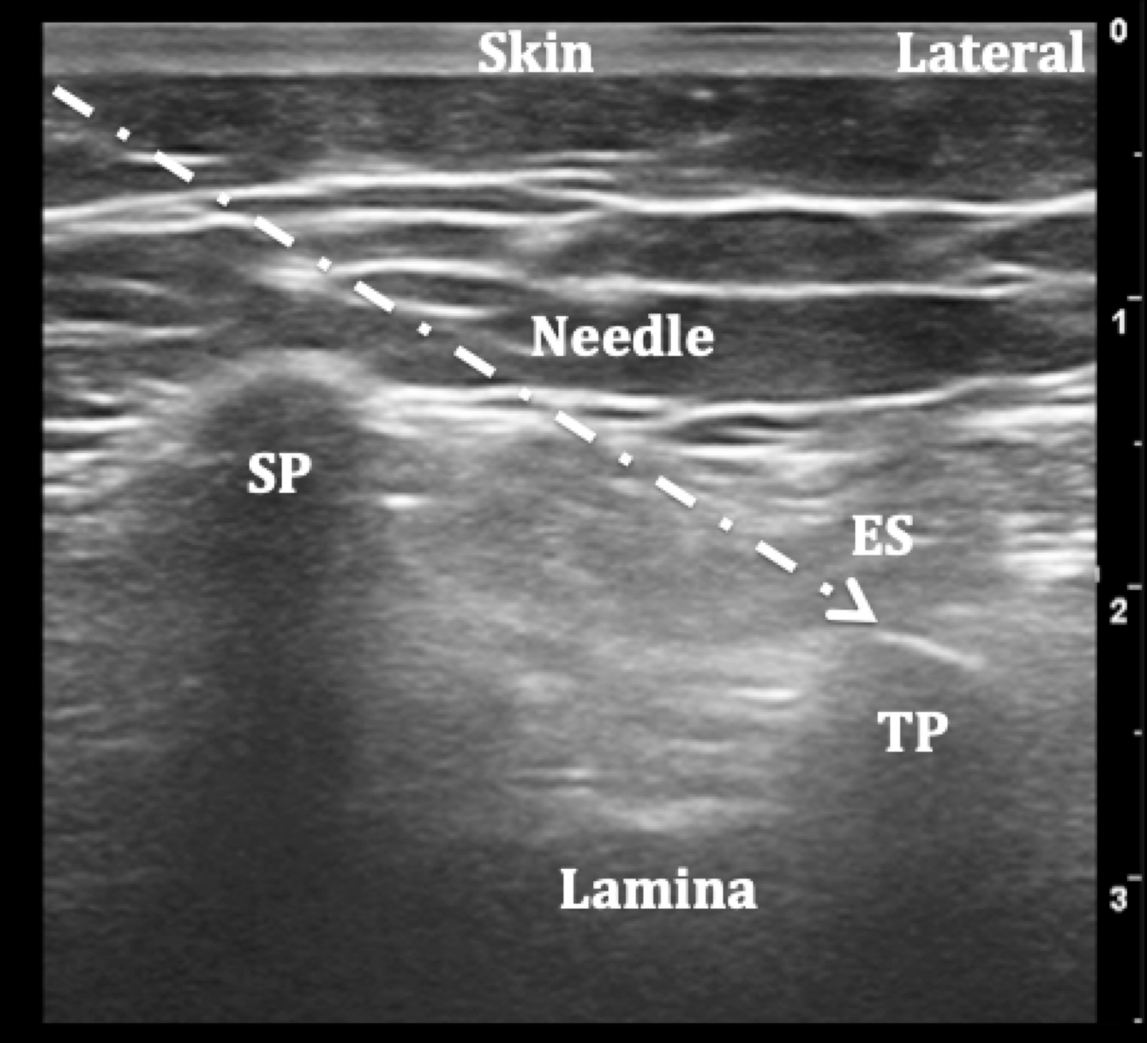
Ultrasound image of the transverse approach to erector spinae plane block (ESPB) SP: Spinous process, ES: Erector spinae, TP: Transverse process.

**Figure 3:**
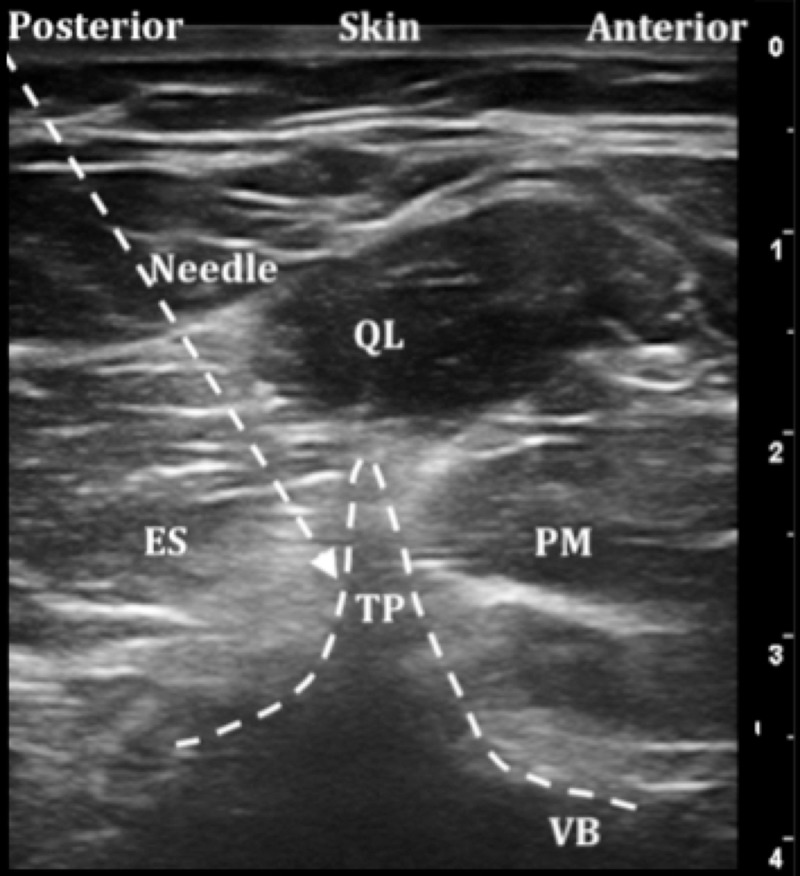
Ultrasound image of the Aksu approach to erector spinae plane block (ESPB) at lumbar levels ES: Erector spinae, QL: Quadratus lumborum muscle, PM: Psoas muscle, TP: Transverse process, VB: Vertebral body.

ESPB was applied using three different techniques: the classic approach as seen in Figure 1, the transverse approach [22] as seen in Figure 2, and the Aksu approach [20] as seen in Figure 3.

Ninety-seven patients underwent ESPB in the prone position and 44 in the lateral decubitus position, the Aksu approach [20] being applied to 35 of these.

Unilateral ESPB was performed on 112 patients, while 29 received a bilateral block. Five of these bilaterally blocked patients were received a sacral ESPB, which was a newly defined level for this block application. Four of these patients were operated for circumcision and one for anoplasty.

ESPB was used for 13 different indications. The mean parental or patient satisfaction score was 9.2 ± 0.65. Indications, block application levels, numbers of patients requiring rescue analgesia, and types of rescue analgesia are shown in Table 1. 

**Table 1:** Indications and levels of erector spinae plane block (ESPB) and rescue analgesic use NSAID: nonsteroidal anti-inflammatory drugs.

	Number of Patients	Application Level	Number of Patients Requiring Rescue Analgesia (Paracetamol / NSAID)
Inguinal hernia repair	50	L1-L2 /Aksu approach	5/0
Orchiopexy	47	L1-L2 /Aksu approach	5/1
Hydrocelectomy	10	L1-L2 /Aksu approach	1/0
Laparoscopic cholecystectomy	6	T7-T8	1/1
Nephrectomy	6	T10-T12	2/0
Varicocelectomy	4	T11-T12	0/1
Ovarian surgery	6	T10	1/3
Thoracic surgery	1	T4	1/0
Breast surgery	2	T4	0/1
Anoplasty	1	S4	0/0
Pyeloplasty	2	T9-T10	1/0
Circumcision	4	S3-S4	0/0
Colostomy surgery	2	T10-T11	2/0

We determined only one exception to the LA dosage. For the patient that underwent thoracotomy, unilateral ESPB was performed from the T4 level with 1 ml/kg 0.25% bupivacaine. No block-related complications were observed in any patients.

## Discussion

This retrospective cohort study investigated ESPB applications for 141 patients with 13 different indications, of which eight were thoracic, three were lumbar, and two were at the sacral level. The use of three different novel techniques reported in this study.

Although ESPB is a relatively new regional anesthesia technique, it has attracted significant interest from clinicians due to its clinical effectiveness and ease of administration. There are increasing reports of its use for different indications. As for many other regional techniques, reports of its use in pediatric patients are still limited and are also restricted to case reports, except for Aksu et al. [7].

Indications for ESPB for postoperative analgesia in pediatric surgeries to date include thoracotomy [8-9], video-assisted thoracoscopic surgery [10], pectus excavatum/carinatum [11], vascular ring repair [12], sternotomy [13], major abdominal surgery [14], laparoscopic cholecystectomy [15-16], nephrectomy [17], pyeloplasty [18], inguinal hernia repair [7,19-21], orchiopexy [7], hydrocelectomy [7], varicocelectomy [22] and hip surgery [23]. There has also been one report of its use in pain management in pediatric palliative care [24]. We have achieved successful applications in five new indications for pediatric patients, ovarian surgery, breast surgery, anoplasty, colostomy, and circumcision. As this interest in ESPB continues to grow, it may be anticipated that many more indications will be identified.

The main concern regarding ESPB is still a lack of knowledge concerning its mechanism of action. There is no consensus in the literature, and controversial results are even being reported, as in the review study by De Cassai et al. [25]. Possible mechanisms suggested to date by cadaveric, anatomic, and magnetic resonance imaging studies include epidural, paravertebral, intercostal, and extensive lateral and longitudinal diffusion [25]. Based on our current data, we think that epidural spread of LA may contribute to analgesic effect significantly, and we would advise clinicians to consider epidural spread when deciding on the application level and the volume of LA. Further data are still needed for a definite conclusion, but regardless of the mechanism of action, the clinical efficacy of ESPB is undeniable.

There may be some technical difficulties in the pediatric population due to anatomical and physiological differences. This patient group has thinner muscle layers, loose connective tissues, and sliding fascial planes. The depth from the skin to the transverse process may also be less than 1 cm, depending on the patient’s age. Placing the needle tip immediately beneath the erector spinae muscle may constitute a significant challenge requiring a fine needling technique and a stable patient position. Even though prone positioning appears to be a better option than the lateral position, another difficulty arises since these patients are commonly under general analgesia. Placing the patient in the prone position and repositioning to the supine position may constitute another risk for airway safety. In the case of lumbar ESPB, we overcome these technical and safety concerns by applying the block with the Aksu approach. For thoracic indications, however, we prefer the prone position over the lateral position.

Two complications of ESPB have been reported to date, pneumothorax, and motor weakness [26-27]. Pneumothorax, in our opinion, should be the most unexpected complication where the transverse process represents a natural anatomical barrier between the pleura and needle. Motor weakness following lumbar or low thoracic ESPB with a high volume of LA may be expected due to a widespread to the lumbar plexus. The LA volume used in our clinic is 0.5 ml/kg, limited to a maximum of 20 ml for pediatric patients. According to our records, no motor weakness or block-related complication has been observed to date.

Our results revealed that ESPB has an opioid-sparing effect in pediatric patients, even in major surgeries. However, in addition to the effectiveness of the block, we also think that standardization was one of the major components of adequate pain management achieved. Components of our standard pain management protocol include administering regional techniques before the start of the surgery, giving analgesics at the end of the surgery, evaluating every child with an appropriate pain scoring system according to its age, and administering analgesics based on the pain ladder. We suggest that clinicians adapt their protocols based on their clinical conditions and experiences.

There were some limitations to this study. First, since the data were collected retrospectively and were limited to the case-based experience of a single-center, our results should now be confirmed by future prospective studies. Another limitation was a lack of knowledge concerning the sensory dermal block area of the ESPB. Since all blocks were performed under general anesthesia, it was difficult to determine the exact dermatomal spread and limits of the sensorial block for this group of patients.

## Conclusions

In conclusion, based on our current clinical experience and understanding obtained from the previous literature, ESPB is a safe and effective procedure for achieving opioid-free postoperative analgesia in many different surgical procedures in pediatric patients. While recommending its routine use for pediatric patients, we think that more prospective randomized clinical trials are now needed for a more accurate conclusion concerning its efficiency and safety, and the application levels and LA doses to be used.
